# Potential Effect of COVID-19 on Maternal and Infant Outcome: Lesson From SARS

**DOI:** 10.3389/fped.2020.00511

**Published:** 2020-08-07

**Authors:** Yun Wang, Yiliang Wang, Xiaoxue Han, Jiazhuo Ye, Ruiman Li

**Affiliations:** ^1^Department of Obstetrics and Gynecology, The First Affiliated Hospital of Jinan University, Guangzhou, China; ^2^Guangzhou Jinan Biomedicine Research and Development Center, College of Life Science and Technology, Jinan University, Guangzhou, China

**Keywords:** COVID-19, SARS-CoV-2, maternal outcome, infant outcome, SARS-CoV

## Abstract

The coronavirus disease 2019 (COVID-19), caused by SARS-CoV-2, is highly infectious and its ongoing outbreak has been declared a global pandemic by the WHO. Pregnant women are susceptible to respiratory pathogens and the development of severe pneumonia, suggesting the urgent need to assess the potential maternal and infant outcome of pregnancy with COVID-19. The intrauterine vertical transmission potential of SARS-CoV-2 also remains controversial. Herein, we discuss the potential effect of COVID-19 on maternal and infant outcomes based on current studies, including those published in Chinese, in a total of 80 mothers with COVID-19 and 80 infants. We also comprehensively explored the mother-to-child transmission routes of SARS-CoV-2, in particular the route of intrauterine vertical transmission. Given SARS-CoV-2 is a sister to SARS-CoV, of the SARS-related coronavirus species, we made a comprehensive comparison between them to learn from experiences with SARS. Although there is no evidence supporting the intrauterine vertical transmission of SARS-CoV-2, our comprehensive analysis suggests that the adverse maternal and infant outcomes caused by COVID-19 cannot be underestimated. Further, we speculated that the inconsistency between nucleic acids and serological characteristics IgM to SARS-CoV-2 of infants' specimens may be caused by the disruption of the amniotic barrier by the inflammatory factors induced by SARS-CoV-2 infection. Our review is beneficial to understand the effect of SARS-CoV-2 on maternal and infant outcomes.

## Introduction

At the beginning of December 2019, a cluster of pneumonia cases with unknown causes were reported (1, 2). A subsequent high-throughput sequencing revealed that the pneumonia epidemic resulted from a novel beta coronavirus tentatively named “2019 novel coronavirus” (2019-nCoV) that was subsequently termed “severe acute respiratory syndrome coronavirus 2” (SARS-CoV-2) (3–5). SARS-CoV-2 is a sister to SARS-CoV, of the SARS-related coronavirus species (4–8). Pneumonia caused by SARS-CoV-2 was correspondingly termed “coronavirus disease 2019” (COVID-19) (4, 5). The ongoing outbreak of COVID-19 has posed a great threat to global human health, which has been declared by the WHO as a global public health emergency (1, 2). As shown by the Center for Systems Science and Engineering at Johns Hopkins University (last updated on 07/07/2020), the global cumulative number of confirmed cases has reached 11,779,263, with 6,758,547 cures, and 540,948 deaths (9). Due to the high transmissibility of COVID-19, the prevention, and control of COVID-19 infection has become a major concern (4, 5). Pregnant women are more susceptible to respiratory pathogens and the development of severe pneumonia than the general population, especially so for those with chronic diseases or maternal complications (4). The physiologic changes in pregnancy, including altered cell-mediated immunity, and alterations in pulmonary function, may confer the susceptibility and severity of pneumonia to pregnant women (6, 10, 11). Pneumonia arising from infectious etiology is the most common non-obstetric infectious condition that occurs in pregnant women (6, 12, 13). In particular, universal SARS-Cov-2 screening for women admitted for delivery found that all women with positive test results were asymptomatic at the time of testing (14, 15). Therefore, the effect of SARS-CoV-2 infection on maternal and infant outcomes needs to be explored, especially the intrauterine vertical transmission potential of COVID-19. Moreover, in the use of a reverse transcriptase-polymerase chain reaction (RT-PCR) and the specific antibody to SARS-CoV-2 of neonate samples remains controversial (4, 5, 16–20). Given SARS-CoV-2 is a sister of SARS-CoV, it is important for us to learn from the experience of preventing and controlling SARS-CoV among pregnant people. In this review, we made a comprehensive comparison of SARS-CoV-2 and SARS-CoV in genetic, infection, transmission, and clinical characteristics. Based on such a comparison, we summarized the potential maternal, and infant outcomes from pregnancy with COVID-19 or SARS. Further, we discuss the potential of mother-to-child transmission of SARS-CoV-2, in particular the possibility of intrauterine vertical transmission. The guidelines for those women with SARS-CoV-2 infections during pregnancy and puerperium prepared by numerous experts were also briefly presented (21). Considering the ongoing global public health emergency, we believe that our review is important for understanding the mother-to-child transmission potential of SARS-CoV-2 and its implication for the safe management of COVID-19 in pregnancy.

## Comparison Between SARS-CoV AND SARS-CoV-2

The pathogen contributing to the COVID-19 epidemic was tentatively named “2019-nCoV” (3, 22). Based on phylogeny, taxonomy, and established practice, the Coronavirus Study Group (CSG) of the International Committee on Taxonomy of Viruses formally recognizes this virus as a sister to SARS-CoV and designates it as SARS-CoV-2 (8). A coronavirus is spherical, enveloped, and the largest of the positive-strand RNA virus and SARS-CoV-2 is the seventh member of enveloped RNA coronaviruses with the ability to infect humans (2, 6). The coronaviruses currently known to infect humans include HCoV-229E and HCoV-NL63 (*Alphacoronavirus* genus), HCoV-OC43, HCoV-HKU1, MERS-CoV, SARS-CoV, and SARS-CoV-2 (*Betacoronavirus* genus) (23). SARS-CoV is the pathogen that caused the SARS epidemic from 2002 to 2003 (24). There were 8,422 cases and 916 deaths in 29 countries, with most of them having occurred in mainland China by 31 July 2003 (24).

Given the great similarity between SARS-CoV and SARS-CoV-2, a comprehensive comparison between these two viruses can help us learn from the SARS epidemic to control and prevent COVID-19. Their comparisons were mainly presented according to their clinical and viral characteristics (Table 1). In general, there was a 79.5% similarity in the whole genome between SARS-CoV and SARS-CoV-2, while only a 74.9% similarity in the gene coding spike glycoprotein (3, 22, 25). Both SARS-CoV-2 and SARS-CoV spread rapidly from human-to-human transmission (7, 28–32). SARS-CoV can be spread via respiratory droplets, secretions, nosocomial contacts, and mechanical aerosols, such as the aerosols arising from the flushing of toilets (33–35). SARS-CoV-2 seems to spread more easily among humans than SARS-CoV, which may result from the various modes of transmission and its high affinity with its receptor angiotensin-converting enzyme 2 (ACE2) (Table 1). The latest pilot experiment confirmed that 4 out of 62 stool specimens (6.5%) tested positive to SARS-CoV-2, and another 4 patients who tested positive toward SARS-CoV-2 in rectal swabs also had SARS-CoV-2 detected in their gastrointestinal tract, saliva, or urine (7). The results suggest the possibility of transmission via aerosols arising from the flushing of toilets. In particular, SARS-CoV-2 can be detected in esophageal erosion and bleeding sites in cases with severe peptic ulcers after symptom onset (7). Although these results only suggest the existence of SARS-CoV-2 nucleotides fragments in these samples, Sun et al. (36) reported that urine samples of COVID-19 patients can isolate SARS-CoV-2 with the infectious ability, suggesting the existence of infectious viral particles in these samples. Moreover, the gastrointestinal tract highly expressed ACE2 as indicated by the Human Protein Atlas, which may explain the existence of SARS-CoV-2 in urine and stool specimens (7). Indeed, the 20–30-fold higher affinity of SARS-CoV-2 spike glycoproteins binding to ACE2 than the SARS-CoV spike protein may also enable the rapid transmission of COVID-19 (25–27). Collectively, the mounting routes of transmission and high affinity with ACE2 might jointly contribute to the rapid spread of SARS-CoV-2. However, COVID-19 exhibited a lower-case fatality rate than SARS (7). The median incubation period of SARS-CoV is also longer than SARS-CoV (7).

**Table 1:** Comparisons between SARS-CoV and SARS-CoV-2.

**Items**	**SARS-CoV**	**SARS-CoV-2**	**References**
First location	Guangdong, China	Wuhan, Hubei, China	(7)
Classification	Genus *Betacoronavirus*, subgenus *Sarbecovirus*	Genus *Betacoronavirus*, subgenus *Sarbecovirus*	(3, 22, 25)
Disease name	SARS	COVID-19	(8, 24)
Clinical characteristics	Fever (99–100%); Cough (62–100%); Diarrhea (20–25%); Chest radiograph abnormality (94–100%); Leukopenia (25–35%); Lymphopenia (68–85%); Thrombocytopenia (40–45%)	Fever (83–98%); Cough (76–82%); Diarrhea (2–3%); Chest radiograph abnormality (76.4%); Leukopenia (5–9%); Lymphopenia (35–63%); Thrombocytopenia (5–12%)	(7)
Case fatality rate (%)	11	1.4–2.1	(7, 24)
Basic reproduction number (R_0_)	2.0–3.0	2.2–2.68	(24, 26)
Median and range of incubation period (days)	4.6 (2.0–14.0)	5.2 (95%CI: 4.1–7.0)	(7, 24)
Receptor of virus spike protein	ACE2	ACE2(With a 20–30-fold higher affinity than SARS-CoV)	(25–27)
Conventional routes of transmission	Respiratory droplets, secretions, nosocomial contact, and mechanical aerosols	Respiratory droplets, direct contact, fomite transmission	(7, 24)
Evidence of supporting Intrauterine vertical transmission	No	No	(4–6)

Despite the high phylogenetic homogeneity between SARS-CoV-2 and SARS-CoV, there are still some clinical characteristics differentiating COVID-19 from SARS. The symptoms of those infected with SARS-CoV have been more common in respiratory out-patient clinics and wards (37, 38). After analyzing the 1,099 COVID-19 patients, Guan et al. (7) found that the typical radiological finding on chest computed tomographies is ground-glass opacity with a ratio of 50.00%. Consistent with previous publications (1, 32, 39), the most common clinical characteristics of COVID-19 are fever (87.9%) and cough (67.7%), but not the gastrointestinal symptom that is more frequently observed in SARS (7). The absence of fever in COVID-19 seems to be more frequent than in SARS-CoV (1%), as fever occurred in only 43.8% of COVID-19 patients on initial presentation (40), implying the limitation of focusing on fever detection in defining surveillance cases (41).

## Maternal and Infant Outcomes from SARS-CoV-Infected Pregnant Women

Although there were relatively few cases of patients infected with SARS during pregnancy based on previous clinical studies and case reports, there were more than 100 cases of SARS-CoV infections that occurred in pregnant women as estimated by the WHO (24). SARS-CoV infection during pregnancy was associated with a risk of adverse maternal and neonatal complications, including intrauterine growth restriction, preterm delivery, spontaneous miscarriage, severe maternal illnesses, such as, admission to the intensive care unit (ICU), renal failure, and disseminated intravascular coagulopathy, and death (4, 6, 13, 42–46). In detail, a case-control study found that the clinical characteristics of SARS in pregnant women were similar to those reported for non-pregnant patients with SARS (47). However, all the pregnant women with SARS required endotracheal intubation, and six were admitted to the ICU, whereas the intubation rate and ICU admission rate in the non-pregnant group was only 17.5 and 12.5%, respectively (47). There were three deaths among pregnant women with SARS, while no deaths occurred in the non-pregnant women with SARS-CoV infection. Both renal failure and disseminated intravascular coagulopathy were developed more frequently in pregnant SARS patients than non-pregnant SARS women (47). Zhang et al. (46) also reported SARS-CoV infections in five primigravids while none of the five infants had virologic evidence of SARS-CoV. In a more detailed case report, Robertson et al. (48) described a 36-year-old pregnant woman with a SARS-CoV infection. Obstetrical ultrasounds revealed a low-lying placenta (placenta previa), but the pregnancy was otherwise normal. The cesarean section was performed at 38 weeks gestation due to the placenta previa and a healthy baby girl was delivered (48, 49). Antibodies against SARS-CoV were tested positive from the maternal serum, umbilical cord blood, and breast milk. No viral RNA was detected in specimens of maternal serum and whole blood, or in swabs from the maternal nasopharynx and rectum, post-delivery placenta, umbilical cord blood, amniotic fluid, and breast milk. However, no clinical specimens were available for testing from the infant in this study (48). Another 38-year-old woman was exposed to SARS-CoV in the same hotel as the aforementioned patients (50). The serum samples taken on days 28 and 64 post-onset of illness tested positive for antibodies against SARS-CoV. Her pregnancy continued and was unremarkable except for developing elevated glucose levels. Due to the preterm rupture of membranes and fetal distress, this patient underwent a cesarean section at 36 weeks gestation and obtained a healthy baby boy. The mother's serum samples at the time of delivery were positive for antibodies against SARS-CoV, but both umbilical cord blood and placenta were negative. Also, breast milk sampled 12 and 30 days after delivery were negative for SARS-CoV antibodies. The specimens, including maternal blood, stool, nasopharynx samples, and umbilical cord blood of the infant, were negative for SARS-CoV RNA. Consistently, the stool samples from the neonate obtained on days-of-life 12 and 30 were negative for SARS-CoV RNA. Yudin et al. (51) reported a 33-year-old pregnant woman who was admitted to the hospital at 31 weeks' gestation due to SARS. Following a 21-day stay in the hospital, the antibody against SARS-CoV tested positive, while she had a normal labor delivery. Together, there were no cases of vertical transmission identified among the pregnant women with SARS-CoV infection (24, 43, 45, 52). However, the effect of SARS on maternal outcomes seems to be associated with the stage of pregnancy when the onset of SARS-CoV occurs (44, 53). Wong et al. (44) found that the SARS-CoV infections present during the first trimester of pregnancy was more likely to cause spontaneous miscarriages, while infections present after 24 weeks of pregnancy developed into delivered preterm.

## Maternal and Infant Outcomes from Pregnant Women With COVID-19

Current research involving pregnancy with COVID-19 were listed in Table 2. Results seems to be inconsistent between antibody-based serological characteristics and RT-PCR-based virologic evidence of infants. Specifically, a retrospective study published in *The Lancet* from (5) reported that the clinical characteristics of SARS-CoV-2 infection in pregnancy were similar to those reported for non-pregnant adults with a SARS-CoV-2 infection. In brief, the typical symptoms, including fever (in seven of nine patients), cough (in four), myalgia (in three), malaise (in two), and sore throat (in two), were observed in these patients, while none of them developed severe COVID-19 pneumonia or died. All patients underwent a cesarean section and their live births had a 1-min Apgar score of 8–9 and a 5-min Apgar score of 9–10 (5). The samples of amniotic fluid, cord blood, neonatal throat swab, and breastmilk samples from six patients tested negative for SARS-CoV-2 (5), suggesting no intrauterine vertical transmission of SARS-CoV-2 in the nine pregnant COVID-19 patients. However, this study enrolled only nine pregnant women with COVID-19, and sample collection was successful in only six infants (5). Another study from Chen et al. reported four pregnant women with COVID-19 (16). All mothers recovered from COVID-19 and had no critical maternal illness, although one mother suffered severe dyspnea after delivery which required respiratory support, and one developed anemia and dyspnea after admission. Of note, none of the three infants whose parents provided consent to be diagnosed tested positive for SARS-CoV-2 from throat swab samples or developed serious clinical symptoms such as fever, cough, or diarrhea. However, two newborns had a rash, which disappeared spontaneously without treatment; a newborn from the mother with placenta previa was considered to suffer from transient tachypnea of the newborn and was supported by non-invasive mechanical ventilation for 3 days. Of note, a study published in *JAMA Pediatrics* indicated three neonates born to a pregnant woman with COVID-19 tested positive for SARS-CoV-2 by qRT-PCR (20). However, as indicated by the medical record, the throat swab sample of the neonate was collected at more than 48 h after delivery. No direct testing of intrauterine tissue samples, such as amniotic fluid, cord blood, or placenta, was collected to detect SARS-CoV-2, which is critical for confirming that the SARS-CoV-2 infection in the neonate was due to intrauterine transmission (20). Therefore, intrauterine SARS-CoV-2 infection remains uncertain.

**Table 2:** Maternal and infant outcome of pregnant women with COVID-19 reported by the indicated study.

**Sample size**	**Pregnancy stage**	**Delivery manner**	**Infant outcome**	**Maternal outcome**	**RT-PCR results of neonate samples for SARS-CoV-2**	**References**
Nine (only six obtained samples successful)	Third trimester (9/9)	Cesarean section (9/9)	Premature (4/9) Increased myocardial enzymes and creatine kinase-myocardial (1/9)	Lymphopenia (5/9) Fetal distress (2/9)	Positive (0/6) (amniotic fluid, cord blood, throat swab)	(5)
Nine (ten neonates, including two twins)	Third trimester (9/9)	Cesarean section (7/9) Vaginal delivery (2/9)	LGA (1/10) SGA (2/10) Fever (2/10) Thrombocytopenia accompanied by abnormal liver function (2/10) Rapid heart rate (1/10) Vomiting (1/10) Pneumothorax (1/10) Death (1/10)	Fetal distress (9/10)	Positive (0/10) (pharyngeal swab)	(54)
Four	Third trimester (4/4)	Cesarean section (3/4) Vaginal delivery (1/4)	Rashes after birth (2/4) Edema from the mother with cholecystitis (1/4) TTN and required nCPAP after birth from mother with placenta previa (1/4)	Reduced fetal movement (1/4) Anemia and dyspnea (1/4) Lymphopenia (2/4) Increased C-response protein (4/4)	Positive (0/4) (throat swab)	(16)
Six	N/A	Cesarean section at third trimester (6/6)	Increased IgM antibody to SARS-CoV-2 concentration (2/6) Increased IgG antibody to SARS-CoV-2 concentration (5/6) Increased IL-6 concentration (6/6)	Increased IgM/IgG antibody to SARS-CoV-2 concentration (5/6)	Positive (0/6) (throat swabs, blood samples)	(19)
One	Third trimester (1/1)	Cesarean section (1/1)	Increased IgM antibody to SARS-CoV-2 concentration (1/1) Increased IgG antibody to SARS-CoV-2 concentration (1/1)	Increased IgM/IgG antibody to SARS-CoV-2 concentration (1/1)	Positive (0/1) (nasopharyngeal swabs)	(17)
Thirty-three	N/A	Cesarean section (26/33) Vaginal delivery (7/33)	N/A	N/A	Positive (3/33) (nasopharyngeal and anal swabs) All tested positive obtained from Cesarean section	(20)
Three	Third trimester (3/3)	Cesarean section (3/3)	Low birth weight (1/3) Fibrin deposition inside and around the villi with local syncytial nodule increases (3/3)	Increased C-response protein (3/3)	Positive (3/3) (throat swabs)	(55)
Seventeen	Third trimester (17/17)	N/A	Headache (1/17)	N/A	Positive (0/17) (oropharyngeal/ nasopharyngeal combination swab)	(14)
One	Third trimester (1/1)	N/A	Intermittent fever, dry cough, headache, and myalgia (1/1)	A dysplastic and multi-cystic right kidney (1/1)	N/A	(56)

*LGA, large-for-gestational-age; SGA, small-for-gestational-age; TTN, transient tachypnea of the newborn; nCPAP, nasal- Continuous Positive Airway Pressure*.

Recently, two studies published in *JAMA* from separate research teams in China reported that three neonates may have acquired SARS-CoV-2 *in utero* from mothers with COVID-19 based on the elevated IgM antibodies to SARS-CoV-2 in neonates (17, 19).

Specifically, the study from Zeng et al. made a retrospectively review for six pregnant women with COVID-19 (19). All these mothers had mild clinical manifestations and performed cesarean deliveries in their third trimester. Of note, all six newborn babies had a normal 1- and 5-min Apgar score and none of them presented any symptoms of COVID-19. However, serological characteristic results indicated that two infants had SARS-CoV-2-specific IgG and IgM concentrations higher than the normal level (<10 AU/mL). Given that IgM is not usually transferred from mother to fetus because of its larger macromolecular structure under normal conditions (57), the author speculated that the neonates may have been infected with SARS-CoV-2 *in utero* from mothers with COVID-19. However, all neonatal throat swabs and blood samples had negative RT-PCR test results. Moreover, this study is limited by the small sample size, lack of cord blood, placenta, amniotic fluid, mother's vaginal secretions, and breast milk and by incomplete information on the outcome of the infants (19). Similar to the case mentioned above, another study from Lan et al. reported that an infant girl born to a mother with COVID-19 (34 weeks, 2 days of gestation) may have acquired SARS-CoV-2 *in utero* due to the elevated IgM antibodies to SARS-CoV-2 (17). However, both the infant's nasopharyngeal swabs and breast milk sampled 3 days after delivery had a negative RT-PCR test result of SARS-CoV-2. Moreover, all neonates had a normal 1- and 5-min Apgar score. The mother's vaginal secretions obtained at delivery also tested negative for SARS-CoV-2. However, this study is limited by the single case, and the lack of amniotic fluid and placenta. There was also no detailed information regarding the pregnancy stage of the onset of COVID-19.

In summary, there was no positive RT-PCR result in the neonate specimens obtained within 24 h post-birth (5, 14, 16, 17, 19), implying no virologic evidence for congenital infection. However, the serological characteristics of infants reported three neonates with elevated IgM antibodies to SARS-CoV-2 born to a mother with COVID-19, suggesting a possible vertical transmission of SARS-CoV-2 from mother to newborn (17, 19). Indeed, the virologic evidence for supporting the utero transmission should be diagnosed based on RT-PCR test results of the samples from neonates but not IgM detection with a high incidence of its false-positive and false-negative results (58, 59). A reasonable explanation for such inconsistency may be the disruption of the placenta or amniotic barrier caused by the inflammatory mediators from mothers that, induced by SARS-CoV-2, facilitates the cross of IgG and IgM. In detail, the placenta is a barrier to viral infection (60). The damage of the placenta by SARS-CoV-2 may represent an important link in the vertical transmission according to the experience from SARS-CoV. The two placentas from women who were recovering from SARS-CoV infection in the third trimester of pregnancy had abnormal weights and pathologies (53). By contrast, in the case of COVID-19, whether the placentas from those pregnant while infected with COVID-19 were abnormal or damaged in most of these studies are unknown (5, 16, 17, 19). Indeed, a study reported that there were various degrees of fibrin deposition inside and around the villi with local syncytial nodule increases in three placentas from those pregnant while infected with COVID-19, especially a placenta with massive infarction (55). However, these three placenta samples tested negative for the nucleic acid of SARS-CoV-2, suggesting no virologic evidence in the placenta (55). However, another study revealed that SARS-CoV-2 invasion of the placenta in a woman with COVID-19 in the second trimester through molecular and immunohistochemical assays and electron microscopy (61). Moreover, the public antibody-protein profiles resident in Human Protein Atlas (HPA) revealed enrichment of the SARS-CoV-2 receptor ACE2 in the placenta and ovary (62). Collectively, the possibility of SARS-CoV-2 infection acquired from the uterus cannot be excluded, highlighting the potential for severe morbidity among pregnant women with COVID-19.

An EDITORIAL published in *JAMA* holds that SARS-CoV-2 can theoretically be transmitted in the uterus, especially given that virus' nucleic acid has been detected in blood samples (59). However, nucleic acids do not represent infectious particles. Indeed, it had been revealed that inflammatory mediators, including IL-6, IL-1β, and TNF-α, cause severe dysfunction of the amniotic barrier via decreasing the expression of tight junctions-associated factors claudin-3 and claudin-4 and inducing apoptosis of the amniotic epithelial cells (63). Of note, IL-6 has prominent pro-inflammatory properties (64). IL-6 was significantly increased in all infants from mothers with COVID-19 (19) and the clinical and immunological features suggested that both the concentration of IL-6 and TNF-α are higher in severe COVID-19 patients than in moderate patients (65). The elevated IgM antibody to SARS-CoV-2 in the blood was not observed in all neonates, which may be associated with the different levels of inflammatory mediators among them. Collectively, in addition to the possibility of false-positive and false-negative results of IgM (59), disruption of the placenta barrier and amniotic barrier caused by inflammatory mediators causing the elevated IgM concentration also needs to be further investigated. However, determination of the level of ordinary IgG but not specific to SARS-CoV-2 in neonate blood would be a crucial indicator explaining the disruption of the placenta and amniotic barrier.

## Potential of Mother-To-Child Transmission of SARS-CoV-2 Based on the General Routes

In general, the routes of mother-to-child transmission of SARS-CoV-2 mainly include intrauterine vertical transmission, birth, or breastfeeding. There is currently no evidence supporting the intrauterine vertical transmission of both SARS-CoV and SARS-CoV-2 based on the discussion above (4–6, 42, 51). However, all the pregnant women recruited in these studies were in their third trimester. Of note, the effect of SARS-CoV-2 on the infant and maternal outcome may be closely associated with their pregnancy stage during the virus infection, which was observed in both SARS-CoV and rubella (44, 66). Therefore, the possibility of intrauterine transmission in pregnancy with SARS-CoV-2 infection in the first or second trimester of pregnancy cannot be overlooked. The potential damage caused by inflammatory factors (above) also needs to be assessed.

For the transmission during birth, most of the people pregnant while infected with COVID-19 discussed above underwent a cesarean section to deliver the live births in current studies, three neonatal from which exhibited early-onset infection with SARS-CoV-2 (20). By contrast, there were ten patients with COVID-19 who performed vaginal delivery, all infants from which tested negative for SARS-CoV-2 (16, 20, 54). Of note, such low transmitted cases were greatly based on the comprehensive protective methods. Indeed, the samples of vaginal mucosa and shedding in birth canals are crucial samples indicating whether SARS-CoV-2 could be transmitted during vaginal delivery. There were few studies that collected vaginal secretion (1/80) or infant blood (12/80); all tested negative for SARS-CoV-2 (5). Further, as revealed by HPA Tissue Atlas, vaginal secretion expresses virtually no ACE2 (62), implying that SARS-CoV-2 may not infect the tissue. Together, the risk of SARS-CoV-2 transmission by vaginal delivery seems low, although more definitive evidence is required.

Finally, to determine the potential of SARS-CoV-2 transmission via breastfeeding, several studies collected and analyzed breast milk samples (7/63) from patients with COVID-19 pneumonia after their first lactation (5, 17). However, these samples tested negative for SARS-CoV-2, suggesting no evidence supporting the breastfeeding transmission of SARS-CoV-2 (5). Of note, such results were similar to pregnancies with SARS-CoV infection. No viral RNA was detected in the specimens of umbilical cord blood, amniotic fluid, and breast milk from those pregnant while infected with SARS-CoV (48–50). Indeed, the antibody against SARS-CoV can be tested from the umbilical cord and breast milk (48–50). Based on such experiences from SARS-CoV, the antibody against SARS-CoV-2 derived from pregnancy may penetrate the placental barrier to orchestrate antiviral defense in the fetus to combat SARS-CoV-2, which needs to be further determined.

## Future Perspective: Management Guidelines for Obstetric Patients and Neonates Born to Mothers With Suspected or Probable COVID-19

In summary, there was a low possibility for mother-to-child transmission of SARS-CoV-2 if adequate protective measures were taken. However, the most crucial point is the potential effect of COVID-19 on maternal and fetal outcomes, rather than whether SARS-CoV-2 can be acquired from the uterus; however, the determination of mother-to-child transmission potential is also important. That said, the effect of COVID-19 on maternal and fetal outcomes should be paid considerable attention. According to the experience from SARS, although no mother-to-child transmission was observed in SARS, SARS-CoV infection was associated with a high risk of severe maternal illness, maternal death, and spontaneous miscarriages (4, 6, 13, 42–46). Indeed, maternal pneumonia is closely associated with a high incidence of various adverse obstetrical outcomes, including the premature rupture of membranes, preterm labor, intrauterine fetal demise, intrauterine growth restriction, and neonatal death (67–69). Further, although observed in a few cases, COVID-19 may be related to the adverse maternal and infant outcome, including premature births, fetal distress, abnormal fetal liver function, rapid heart rate, etc. (Table 2).

To address the safety issues for the obstetrical management and delivery of pregnant women with COVID-19, the advice for those women with SARS-CoV-2 infections during pregnancy and puerperium was prepared by numerous experts from the fields of obstetrics and gynecology, pediatrics, infectious diseases, and critical care (21, 70–75). Similar to the recommendations for the non-pregnant, early isolation, early diagnosis, and early management are still the core criteria of prevention and control transmission for pregnant women with suspected and probable SARS-CoV-2 infection. These recommendations mainly include:

1. At times of COVID-19 outbreaks, all pregnant patients should be assessed for travel history or contact with people from the worst-hit areas of the epidemic within 2 weeks. The definition of a case with suspected COVID-19 should be focused on the clinical symptoms of COVID-19;
2. Pregnant women with labor-confirmed SARS-CoV-2 infection should be treated centrally according to the designation by the department of medical administration. The corresponding risk of adverse pregnancy outcomes contributed by COVID-19 should be informed to the patients;
3. A chest radiograph, especially the computed tomography, is crucial for evaluating the development of COVID-19;
4. Pregnant women with suspected or probable COVID-19 should be informed to the CDC and placed in an isolation room or a negative pressure room if it is available;
5. Prenatal examination and delivery of pregnant women with a SARS-CoV-2 infection should be carried out in negative pressure isolation or on an isolation ward. The management medical staff should wear protective equipment;
6. The timing of childbirth should be based on the specific conditions of the mother and child, the gestational week, and the childbirth conditions. The delivery mode depends on obstetric indication;
7. The specific anesthesia method for SARS-CoV-2-infected pregnant women who require surgical delivery can be general anesthesia and regional anesthesia, which should be performed based on the professional anesthesiologist;
8. Given that the possibility of the intrauterine vertical transmission of SARS-CoV-2 cannot be excluded, all newborns from pregnant patients with suspected or confirmed COVID-19 should be isolated for at least 14 days and should not be breastfed during this period until a SARS-CoV-2 infection is ruled out or cured. The mothers should squeeze milk regularly to ensure lactation. An expert team consisting of obstetricians, nurses, pediatricians, infection control specialists, respiratory therapists, and anesthesiologists should jointly manage pregnant women with COVID-19 and their newborn baby;
9. Pregnant women with COVID-19 should be managed by fixed staff, including obstetrics, neonatal, and other related professionals. The healthcare workers caring for pregnant COVID-19 patients should not care for other patients. All healthcare workers should be daily monitored for fever and cough symptoms of COVID-19. Such individuals should be isolated if they were confirmed or suspected of COVID-19;
10. All health care personnel, trainees, and support staff should be trained in infection control management and containment to prevent the spread of SARS-CoV-2.

## Author Contributions

YuW, YiW, and JY: conception and design, collection and/or assembly of references, data analysis, interpretation, and manuscript writing. XH and RL: conception and design, manuscript writing, and final approval of manuscript. All authors read and approved the final manuscript.

## Conflict of Interest

The authors declare that the research was conducted in the absence of any commercial or financial relationships that could be construed as a potential conflict of interest.

## Abbreviations

SARS-CoV-2: severe acute respiratory syndrome coronavirus 2
SARS-CoV: severe acute respiratory syndrome coronavirus 1
SARS: severe acute respiratory syndrome
COVID-19: 2019 novel coronavirus disease
ACE2: angiotensin-converting enzyme 2
LGA: large-for-gestational-age
SGA: small-for-gestational-age
TTN: transient tachypnea of the newborn
nCPAP: nasal- Continuous Positive Airway Pressure.

## References

[1] Huang C Wang Y Li X Ren L Zhao J Hu Y. Clinical features of patients infected with 2019 novel coronavirus in Wuhan, China. Lancet. (2020) 395:809–15. 10.1016/S0140-6736(20)30183-5 31986264 PMC7159299

[2] Zhu N Zhang D Wang W Li X Yang B Song J. A novel coronavirus from patients with pneumonia in china, 2019. N Engl J Med. (2020) 382:727–33. 10.1056/NEJMoa2001017 31978945 PMC7092803

[3] Lu R Zhao X Li J Niu P Yang B Wu H. Genomic characterisation and epidemiology of 2019 novel coronavirus: implications for virus origins and receptor binding. Lancet. (2020) 395:565–74. 10.1016/S0140-6736(20)30251-8 32007145 PMC7159086

[4] Qiao J. What are the risks of COVID-19 infection in pregnant women? Lancet. (2020) 395:760–2. 10.1016/S0140-6736(20)30365-2 32151334 PMC7158939

[5] Chen H Guo J Wang C Luo F Yu X Zhang W. Clinical characteristics and intrauterine vertical transmission potential of COVID-19 infection in nine pregnant women: a retrospective review of medical records. Lancet. (2020) 395:809–15. 10.1016/S0140-6736(20)30360-3 32151335 PMC7159281

[6] Schwartz D Graham A. Potential maternal and infant outcomes from (Wuhan) coronavirus 2019-nCoV infecting pregnant women: lessons from SARS, MERS, and other human coronavirus infections. Viruses. (2020) 12:194. 10.3390/v12020194 32050635 PMC7077337

[7] Guan WJ Ni ZY Hu Y Liang WH Ou CQ He JX Clinical characteristics of 2019 novel coronavirus infection in China. N Engl J Med. (2020) 382:1708–20. 10.1101/2020.02.06.20020974 32109013 PMC7092819

[8] Coronaviridae Study Group of the International Committee on Taxonomy of Viruses. The species Severe acute respiratory syndrome-related coronavirus: classifying 2019-nCoV and naming it SARS-CoV-2. Nat Microbiol. (2020) 5:536–44. 10.1038/s41564-020-0695-z 32123347 PMC7095448

[9] Dong E Du H Gardner L. An interactive web-based dashboard to track COVID-19 in real time. Lancet Infect Dis. (2020) 20:533–4. 10.1016/S1473-3099(20)30120-1 32087114 PMC7159018

[10] Harris J Influenza occurring in pregnant women: a statistical study of thirteen hundred and fifty cases. J Am Med Assoc. (1919) 72:978–80. 10.1001/jama.1919.02610140008002

[11] Eickhoff T Sherman I Serfling R. Observations on excess mortality associated with epidemic influenza. Md State Med J. (1962) 11:115–6. 13889578

[12] Visscher H Visscher R. Indirect obstetric deaths in the state of Michigan 1960-1968. Am J Obstet Gynecol. (1971) 109:1187–96. 10.1016/0002-9378(71)90664-8 5554850

[13] Jamieson D Theiler R Rasmussen S Emerging infections and pregnancy. Emerg Infect Dis. (2006) 12:1638–43. 10.3201/eid1211.060152 17283611 PMC3372330

[14] Fassett MJ Lurvey LD Yasumura L Nguyen M Colli JJ Volodarskiy M. Universal SARS-Cov-2 screening in women admitted for delivery in a large managed care organization. Am J Perinatol. (2020). 10.1055/s-0040-1714060 32620022 PMC7516390

[15] Sutton D Fuchs K D'Alton M Goffman D. Universal screening for SARS-CoV-2 in women admitted for delivery. N Engl J Med. (2020) 382:2163–4. 10.1056/NEJMc2009316 32283004 PMC7175422

[16] Chen Y Peng H Wang L Zhao Y Zeng L Gao H. Infants born to mothers with a new coronavirus (COVID-19). Front Pediatr. (2020) 8:104. 10.3389/fped.2020.00104 32266184 PMC7098456

[17] Dong L Tian J He S Zhu C Wang J Liu C. Possible vertical transmission of SARS-CoV-2 from an infected mother to her newborn. JAMA. (2020) 323:1846–8. 10.1001/jama.2020.4621 32215581 PMC7099527

[18] Li N Han L Peng M Lv Y Ouyang Y Liu K Maternal and neonatal outcomes of pregnant women with COVID-19 pneumonia: a case-control study. Clin Infect Dis. (2020). 10.1101/2020.03.10.20033605 PMC7184430 32249918

[19] Zeng H Xu C Fan J Tang Y Deng Q Zhang W. Antibodies in infants born to mothers with COVID-19 pneumonia. JAMA. (2020) 323:1848–49. 10.1001/jama.2020.4861 32215589 PMC7099444

[20] Zeng L Xia S Yuan W Yan K Xiao F Shao J. Neonatal early-onset infection with SARS-CoV-2 in 33 neonates born to mothers with COVID-19 in Wuhan, China. JAMA Pediatr. (2020) 174:722–5. 10.1001/jamapediatrics.2020.0878 32215598 PMC7099530

[21] Wang Y Jin F Wang R Li F Wu Y Kitazato K. HSP90: a promising broad-spectrum antiviral drug target. Arch Virol. (2017) 162:3269–82. 10.1007/s00705-017-3511-1 28780632

[22] Wu F Zhao S Yu B Chen Y Wang W Song ZG. A new coronavirus associated with human respiratory disease in China. Nature. (2020) 579:265–9. 10.1038/s41586-020-2008-3 32015508 PMC7094943

[23] Hui D. Epidemic and emerging coronaviruses (severe acute respiratory syndrome and middle east respiratory syndrome). Clin Chest Med. (2016) 38:71–86. 10.1016/j.ccm.2016.11.007 28159163 PMC7131795

[24] WHO Consensus Document on the Epidemiology of Severe Acute Respiratory Syndrome (SARS). Geneva: World Health Organization (2003).

[25] Zhou P Yang X Wang XG Hu B Zhang L Zhang W. A pneumonia outbreak associated with a new coronavirus of probable bat origin. Nature. (2020) 579:270–3. 10.1038/s41586-020-2012-7 32015507 PMC7095418

[26] Wrapp D Wang N Corbett KS Goldsmith JA Hsieh CL Abiona O. Cryo-EM structure of the 2019-nCoV spike in the prefusion conformation. Science. (2020) 367:1260–3. 10.1126/science.abb2507 32075877 PMC7164637

[27] Xu X Chen P Wang J Feng J Zhou H Li X. Evolution of the novel coronavirus from the ongoing Wuhan outbreak and modeling of its spike protein for risk of human transmission. Sci China Life Sci. (2020) 63:457–60. 10.1007/s11427-020-1637-5 32009228 PMC7089049

[28] Chan J Yuan S Kok KH To K Chu H Yang J. A familial cluster of pneumonia associated with the 2019 novel coronavirus indicating person-to-person transmission: a study of a family cluster. Lancet. (2020) 395:514–23. 10.1016/S0140-6736(20)30154-9 31986261 PMC7159286

[29] Phan L Nguyen T Luong Q Nguyen T Nguyen H Le H. Importation and human-to-human transmission of a novel coronavirus in Vietnam. N Engl J Med. (2020) 382:872–4. 10.1056/NEJMc2001272 31991079 PMC7121428

[30] Rothe C Schunk M Sothmann P Bretzel G Froeschl G Wallrauch C. Transmission of 2019-nCoV infection from an asymptomatic contact in germany. N Engl J Med. (2020) 382:970–1. 10.1056/NEJMc2001468 32003551 PMC7120970

[31] Wu J Leung K Leung G. Nowcasting and forecasting the potential domestic and international spread of the 2019-nCoV outbreak originating in Wuhan, China: a modelling study. Lancet. (2020) 395:689–97. 10.1016/S0140-6736(20)30260-9 32014114 PMC7159271

[32] Li Q Guan X Wu P Wang X Zhou L Tong Y. Early transmission dynamics in Wuhan, China, of novel coronavirus-infected pneumonia. N Engl J Med. (2020) 382:1199–207. 10.1056/NEJMoa2001316 31995857 PMC7121484

[33] Poutanen S Low D Henry B Finkelstein S Rose D Green K. Identification of severe acute respiratory syndrome in Canada. N Engl J Med. (2003) 348:1995–2005. 10.1056/NEJMoa030634 12671061

[34] Seto WH Tsang D Yung R Ching TY Ng T Ho M. Effectiveness of precautions against droplets and contact in prevention of nosocomial transmission of severe acute respiratory syndrome (SARS). Lancet. (2003) 361:1519–20. 10.1016/S0140-6736(03)13168-6 12737864 PMC7112437

[35] Hung L. The SARS Epidemic in Hong Kong—what lessons have we learned? J R Soc Med. (2003) 96:374–8. 10.1258/jrsm.96.8.374 12893851 PMC539564

[36] Sun J Zhu A Li H Zheng K Zhuang Z Chen Z. Isolation of infectious SARS-CoV-2 from urine of a COVID-19 patient. Emerg Microbes Infect. (2020) 9:991–3. 10.1080/22221751.2020.1760144 32342724 PMC7301718

[37] Leung W To KF Chan P Chan H Wu A Lee N. Enteric involvement of severe acute respiratory syndrome - Associated coronavirus infection. Gastroenterology. (2003) 125:1011–7. 10.1016/S0016-5085(03)01215-0 14517783 PMC7126982

[38] Minodier L Charrel R Ceccaldi PE Werf S Blanchon T Hanslik T. Prevalence of gastrointestinal symptoms in patients with influenza, clinical significance, and pathophysiology of human influenza viruses in faecal samples: what do we know? Virol J. (2015) 12:215. 10.1186/s12985-015-0448-4 26651485 PMC4676820

[39] Chen N Zhou M Dong X Qu J Gong F Han Y. Epidemiological and clinical characteristics of 99 cases of 2019 novel coronavirus pneumonia in Wuhan, China: a descriptive study. Lancet. (2020) 395:507–13. 10.1016/S0140-6736(20)30211-7 32007143 PMC7135076

[40] Zumla A Hui D Perlman S. Middle east respiratory syndrome. Lancet. (2015) 386:995–1007. 10.1016/S0140-6736(15)60454-8 26049252 PMC4721578

[41] WHO Clinical Management of Severe Acute Respiratory Infection When Novel Coronavirus (nCoV) Infection is Suspected: Interim Guidance. Geneva: WHO (2020).

[42] Wong S Chow KM Swiet M. Severe acute respiratory syndrome and pregnancy. BJOG Int J Obstet Gynaecol. (2003) 110:641–2. 10.1046/j.1471-0528.2003.03008.x 12842052 PMC7161769

[43] Ng P So KW Leung TF Cheng F Lyon D Wong W. Infection control for SARS in a tertiary neonatal centre. Arch Dis Childh Fetal Neonatal Ed. (2003) 88:F405–9. 10.1136/fn.88.5.F405 12937045 PMC1721604

[44] Wong S Chow K Leung T Ng W Ng T Shek CC. Pregnancy and perinatal outcomes of women with severe acute respiratory syndrome. Am J Obstet Gynecol. (2004) 191:292–7. 10.1016/j.ajog.2003.11.019 15295381 PMC7137614

[45] Ng P Leung C Chiu WK Wong S Hon EKL. SARS in newborns and children. Biol Neonate. (2004) 85:293–8. 10.1159/000078174 15218286

[46] Zhang JP Wang YH Chen LN Zhang R Xie YF. Clinical analysis of pregnancy in second and third trimesters complicated severe acute respiratory syndrome. Zhonghua fu chan ke za zhi. (2003) 38:516–20. 14521763

[47] Lam C Wong S Leung T Chow K Yu W Wong T. A case-controlled study comparing clinical course and outcomes of pregnant and non-pregnant women with severe acute respiratory syndrome. BJOG Int J Obstet Gynaecol. (2004) 111:771–4. 10.1111/j.1471-0528.2004.00199.x 15270922 PMC7161819

[48] Robertson CA Lowther SA Birch T Tan C Sorhage F Stockman L. SARS and pregnancy: a case report. Emerg Infect Dis. (2004) 10:345–8. 10.3201/eid1002.030736 15030710 PMC3322896

[49] Schneider E Duncan D Reiken M Perry R Messick J Sheedy C. SARS in pregnancy. AWHONN Lifelines. (2004) 8:122–8. 10.1177/1091592304265557 15137260 PMC7110542

[50] Stockman L Lowther S Coy K Saw J Parashar U. SARS during pregnancy, United States. Emerg Infect Dis. (2004) 10:1689–90. 10.3201/eid1009.040244 15503406 PMC3320293

[51] Yudin M Steele D Sgro M Read S Kopplin P Gough K. Severe acute respiratory syndrome in pregnancy. Obstet Gynecol. (2005) 105:124–7. 10.1097/01.AOG.0000151598.49129.de 15625153

[52] Shek CC Ng PC Fung GP Cheng FW Chan PK Peiris MJ. Infants born to mothers with severe acute respiratory syndrome. Pediatrics. (2003) 112:e254. 10.1542/peds.112.4.e254 14523207

[53] Ng WF Wong SF Lam A Mak YF Yao H Lee KC. The placentas of patients with severe acute respiratory syndrome: a pathophysiological evaluation. Pathology. (2006) 38:210–8. 10.1080/00313020600696280 16753741 PMC7131423

[54] Zhu H Wang L Fang C Peng S Zhang L Chang G. Clinical analysis of 10 neonates born to mothers with 2019-nCoV pneumonia. Transl Pediatr. (2020) 9:51–60. 10.21037/tp.2020.02.06 32154135 PMC7036645

[55] Wang Y Huang L Wang Y Luo W Li F Xiao J. Single-cell RNA-sequencing analysis identifies host long noncoding RNA MAMDC2-AS1 as a co-factor for HSV-1 nuclear transport. Int J Biol Sci. (2020) 16:1586–603. 10.7150/ijbs.42556 32226304 PMC7097924

[56] Zambrano LI Fuentes-Barahona IC Bejarano-Torres DA Bustillo C Gonzales G Vallecillo-Chinchilla G. A pregnant woman with COVID-19 in Central America. Travel Med Infect Dis. (2020) 101639. 10.1016/j.tmaid.2020.101639 32222420 PMC7271224

[57] Woo PC Lau SK Wong BH Tsoi HW Fung AM Chan KH. Detection of specific antibodies to severe acute respiratory syndrome (SARS) coronavirus nucleocapsid protein for serodiagnosis of SARS coronavirus pneumonia. J Clin Microbiol. (2004) 42:2306–9. 10.1128/JCM.42.5.2306-2309.2004 15131220 PMC404667

[58] Nielsen CM Hansen K Andersen HMK Gerstoft J Vestergaard BF. An enzyme labelled nuclear antigen immunoassay for detection of cytomegalovirus IgM antibodies in human serum: specific and non-specific reactions. J Med Virol. (1987) 22:67–76. 10.1002/jmv.1890220109 3035081

[59] Kimberlin DW Stagno S. Can SARS-CoV-2 Infection Be Acquired *In Utero*? JAMA. (2020) 323:1788–9. 10.1001/jama.2020.4868 32215579

[60] Delorme-Axford E Sadovsky Y Coyne CB. The placenta as a barrier to viral infections. Annu Rev Virol. (2014) 1:133–46. 10.1146/annurev-virology-031413-085524 26958718

[61] Hosier H Farhadian SF Morotti RA Deshmukh U Lu-Culligan A Campbell KH. SARS-CoV-2 infection of the placenta. J Clin Invest. (2020) 139569. 10.1172/JCI139569. [Epub ahead of print]. 32573498 PMC7456249

[62] Wang Y Wang Y Luo W Huang L Xiao J Li F. A comprehensive investigation of the mRNA and protein level of ACE2, the putative receptor of SARS-CoV-2, in human tissues and blood cells. Int J Med Sci. (2020) 17:1522–31. 10.7150/ijms.46695 32669955 PMC7359402

[63] Ross MG. Inflammatory mediators weaken the amniotic membrane barrier through disruption of tight junctions. J Physiol. (2011) 589:5. 10.1113/jphysiol.2010.202903 21224246 PMC3039253

[64] Moore JB June CH. Cytokine release syndrome in severe COVID-19. Science. (2020) 368:473–74. 10.1126/science.abb8925 32303591

[65] Chen G Wu D Guo W Cao Y Huang D Wang H. Clinical and immunological features of severe and moderate coronavirus disease 2019. J Clin Invest. (2020) 130:2620–9. 10.1172/JCI137244 32217835 PMC7190990

[66] Bouthry E Picone O Hamdi G Grangeot-Keros L Ayoubi JM Vauloup-Fellous C. Rubella and pregnancy: diagnosis, management and outcomes. Prenat Diagn. (2014) 34:1246–53. 10.1002/pd.4467 25066688

[67] Benedetti T Valle R Ledger W. Antepartum pneumonia in pregnancy. Am J Obstet Gynecol. (1982) 144:413–7. 10.1016/0002-9378(82)90246-0 7124859

[68] Berkowitz K LaSala A. Risk factors associated with the increasing prevalence of pneumonia during pregnancy. Am J Obstet Gynecol. (1990) 163:981–5. 10.1016/0002-9378(90)91109-P 2403178

[69] Madinger N Greenspoon J Ellrodt A. Pneumonia during pregnancy: has modern technology improved maternal and fetal outcome? Am J Obstet Gynecol. (1989) 161:657–62. 10.1016/0002-9378(89)90373-6 2782348

[70] Liang H Acharya G. Novel corona virus disease (COVID-19) in pregnancy: what clinical recommendations to follow? Acta Obstet Gynecol Scand. (2020) 99:439–42. 10.1111/aogs.13836 32141062

[71] Qi H Luo X Zheng Y Zhang H Li J Zou L. Safe delivery for COVID-19 infected pregnancies. BJOG. (2020) 127:927–9. 10.1111/1471-0528.16231 32219995

[72] Amatya S Corr TE Gandhi CK Glass KM Kresch MJ Mujsce DJ Management of newborns exposed to mothers with confirmed or suspected COVID-19. J Perinatol. (2020) 40:987–96. 10.1038/s41372-020-0695-0 32439956 PMC7241067

[73] Donders F Lonnee-Hoffmann R Tsiakalos A Mendling W Martinez de Oliveira J Judlin P. ISIDOG recommendations concerning COVID-19 and pregnancy. Diagnostics. (2020) 10:243. 10.3390/diagnostics10040243 32338645 PMC7235990

[74] Almudeer A Alallah J AlSaedi S Anabrees J Kattan A AlSalam Z Recommendations for the management of newborn with suspected or confirmed coronavirus disease-19. J Clin Neonatol. (2020) 9:93 10.4103/jcn.JCN_34_20

[75] Bezhenar V Aylamazyan EK Zazerskaya IE Ivanov DO Arakelyan BV Bautin A BRIEF CLINICAL RECOMMENDATIONS: management tactics for pregnant women and women in labor and childbirth with suspected or confirmed COVID-19 infection. (2020). 10.17816/JOWDS20201

